# Individual heterogeneity in fitness in a long‐lived herbivore

**DOI:** 10.1002/ece3.8197

**Published:** 2021-09-30

**Authors:** Madeleine G. Lohman, Thomas V. Riecke, Perry J. Williams, James S. Sedinger

**Affiliations:** ^1^ Program in Ecology, Evolution, and Conservation Biology University of Nevada, Reno Reno Nevada USA; ^2^ Swiss Ornithological Institute Sempach Switzerland; ^3^ Department of Natural Resources and Environmental Science University of Nevada, Reno Reno Nevada USA

**Keywords:** black brant, breeding probability, fitness, life‐history, reproduction, survival, trade‐offs

## Abstract

Heterogeneity in the intrinsic quality and nutritional condition of individuals affects reproductive success and consequently fitness. Black brant (*Branta bernicla nigricans*) are long‐lived, migratory, specialist herbivores. Long migratory pathways and short summer breeding seasons constrain the time and energy available for reproduction, thus magnifying life‐history trade‐offs. These constraints, combined with long lifespans and trade‐offs between current and future reproductive value, provide a model system to examine the role of individual heterogeneity in driving life‐history strategies and individual heterogeneity in fitness. We used hierarchical Bayesian models to examine reproductive trade‐offs, modeling the relationships between within‐year measures of reproductive energy allocation and among‐year demographic rates of individual females breeding on the Yukon‐Kuskokwim Delta, Alaska, using capture–recapture and reproductive data from 1988 to 2014. We generally found that annual survival tended to be buffered against variation in reproductive investment, while breeding probability varied considerably over the range of clutch size‐laying date combinations. We provide evidence for relationships between breeding probability and clutch size, breeding probability and nest initiation date, and an interaction between clutch size and initiation date. Average lifetime clutch size also had a weak positive relationship with apparent survival probability. Our results support the use of demographic buffering strategies for black brant. These results also indirectly suggest associations among environmental conditions during growth, fitness, and energy allocation, highlighting the effects of early growth conditions on individual heterogeneity, and subsequently, lifetime reproductive investment.

## Graphical abstract

Understanding differences in energy allocation toward survival and reproduction within and among years might help explain variability in individual fitness. We used hierarchical Bayesian models to examine reproductive trade‐offs, modeling the relationships between within‐year measures of reproductive energy allocation and among‐year demographic rates of individual females breeding on the Yukon‐Kuskokwim Delta, Alaska, using capture–recapture and reproductive data from 1988 to 2014. Our results support the use of demographic buffering strategies for black brant; reductions in reproductive energy allocation preserve high adult survival rates during years with poor environmental conditions, maximizing future reproductive value.

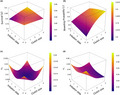

## 1 INTRODUCTION

Expression of life‐history traits may be conditional on the current physiological state or environmental conditions experienced by individuals because such flexibility maximizes fitness (McNamara & Houston, 1996) and the common positive association between fitness traits (van Noordwijk & de Jong, 1986) reflects individual variation in state within populations. The negative association between clutch size and laying date, which is widespread in birds (Klomp, 1970), is an important example of physiological state‐dependent life histories. Covariation between clutch size and laying date is driven by a trade‐off between the quantity of offspring individuals in a particular state can produce on a given breeding date against the declining quality of those offspring as laying date progresses (Daan et al., 1990; Drent & Daan, 1980; Rowe et al., 1994). This covariation is at least partially under genetic control (Sheldon et al., 2003) and mediated by physiological processes that translate environmental cues into the expression of life‐history traits (Meijer et al., 1990; Sinervo & Svensson, 1998). Parallel patterns of clutch size and laying date occur in capital breeders, driven by variation in nutritional status before and during spring migration (Bêty et al., 2003; Prop & de Vries, 1993). In these species, nutritional status influences the timing of migration and arrival on breeding areas. Individuals with smaller nutrient reserves tend to arrive on breeding areas later and nest later (Bêty et al., 2003; Prop et al., 2003), although they do not delay long enough to acquire sufficient nutrients to produce the largest clutches, resulting in a seasonal decline in clutch size (Bêty et al., 2003; Dalhaug et al., 1996; Hamann & Cooke, 1989; Verhulst & Nilsson, 2008). The “individual optimization” or “prudent parent” strategy, as this strategy is known, does not equalize yearly reproductive fitness advantages for individuals in inferior and superior states before the breeding season. Individuals in superior states still produce more and higher quality offspring than those in inferior states before breeding (Bêty et al., 2003; Prop et al., 2003), but individuals along a gradient of state quality each maximize their reproductive fitness conditional on their nutritional state.

Reproductive strategies also derive from trade‐offs between current and future reproductive fitness or the quantity and quality of offspring in the current reproductive event (Stearns, 1992). Trade‐offs between quantity and quality of offspring are well established by hundreds of observational and experimental studies for species with altricial young, beginning with Lack's revolutionary studies (Lack, 1948, 1950; Perrins, 1964) and continuing to the present (Leach et al., 2019). A larger number of studies have detected trade‐offs between quantity and quality of offspring (Klomp, 1970) than between current and future reproduction (Santos & Nakagawa, 2012), possibly because of the difficulty of detecting the latter. Quantity–quality trade‐offs can be assessed using data from a single field season. In contrast, trade‐offs with future reproduction require long‐term studies with marked individuals.

Bird species with precocial young do not feed their offspring, which has led investigators to assume that clutch size must be limited by the proximal constraint of nutrients available to females before or during egg laying (Alisauskas & Ankney, 1992; Ankney & MacInnes, 1978; Lack, 1967). Recently, experimental manipulations of clutch and brood size in black brant (*Branta bernicla nigricans*, hereafter brant), which have precocial young, demonstrated diminishing fitness returns as brood size increased (Sedinger et al., 2017) and costs to future reproduction of producing broods larger than the most common brood size of four (Leach et al., 2019). Additionally, egg size has a positive association with the size of goslings at fledging (Acevedo et al., 2020), which strongly influences first‐year survival (Sedinger & Chelgren, 2007) and recruitment into the breeding population (Riecke et al., 2018; Sedinger et al., 2004). When nutrient reserves are limited, variation in egg size among females (Flint & Sedinger, 1992; Lemons et al., 2011) results from individual trade‐offs between the quantity and quality of offspring produced (Williams, 2001).

Positive covariance in fitness traits among individuals may diminish the ability of researchers to assess trade‐offs at the population level (van Noordwijk & de Jong, 1986; Verhulst & Nilsson, 2008). For example, offspring quality and quantity might be positively correlated in a study with unmanipulated broods because “higher quality” parents produce both more and higher quality offspring. Thus, the ability to assess trade‐offs between fitness traits will depend on whether there is sufficient residual variance remaining after accounting for the relationship between the main effects of the traits of interest. We note that in brant substantial residual variability remains in clutch size after accounting its association with laying date. Sedinger et al. (1995) reported positive correlations among multiple traits associated with fitness, including the probability of breeding, and while not linked to other traits, Lindberg et al. (2013) identified heterogeneity in innate mortality risk. Because researchers could link these traits to growth conditions experienced by individuals, Sedinger, Flint, et al. (1995) attributed much of the variation in fitness to spatial and temporal variability in habitat quality during postnatal growth.

Given these relationships and the importance of understanding life‐history trade‐offs, our objective was to assess the potential for relationships between reproductive traits associated with annual fitness and longer‐term life‐history traits. In this manuscript, we model the relationships between clutch size and nest initiation date and their interaction, and apparent annual survival of breeding females and breeding probabilities. Our expectation was that individuals producing larger and earlier clutches, being on average of higher quality, would experience higher annual survival and breed more frequently than individuals producing smaller and later clutches. We further expected that buffering of life‐history traits would constrain variation in annual survival relative to that of breeding probability. Finally, incorporating an interaction between laying date and clutch size into models of annual survival and breeding probability allowed us to explore the nature of the relationships among the suite of variables away from the mean clutch size‐laying date relationship. For example, we expect that individuals producing clutches larger than expected might experience greater breeding probability associated with positive effects of large families on social status of geese in winter (Poisbleau et al., 2006; Raveling, 1970). We discuss the direct and indirect effects of these relationships on fitness and population dynamics.

## 2 MATERIALS AND METHODS

We collected data at the Tutakoke River Colony (TRC; Sedinger et al., 1993; 61.25°N, 165.61°W) and related brood rearing areas on the YKD near the mouth of the Kashunuk River from 1988 to 2014 (Lindberg & Sedinger, 1998; Sedinger, Flint, et al., 1995). We divided the breeding season into three secondary occasions for a robust design capture–mark–recapture analysis (Kendall & Nichols, 1995). First (May–June), nests were monitored in forty‐nine 50‐m‐radius random plots every four days throughout nest initiation, and again before and during hatch. Observers recorded clutch sizes and initiation dates of nests during this time. Incubation time is generally 23–29 days, varying with laying date and clutch size (Eichholz & Sedinger, 1998). Thus, we back‐calculated initiation dates for hatched nests using mean incubation period (~26 days; Eichholz & Sedinger, 1998, Leach et al., 2017). We also monitored nests of marked individuals outside of plots during this time. Second (June–early July), following hatch, observers entered observation towers to observe marked adults and broods (Sedinger et al., 2001). Third (mid‐late July), adult and juvenile brant were herded into pens and marked with a unique U.S. Geological Survey metal band and an alpha‐numerically coded plastic band during the adult wing‐molt (Sedinger et al., 1997). We included data only for marked adult females (*n* = 7845) to estimate mean annual survival and breeding probability and temporal variation in those parameters because we were interested only in female reproductive trade‐offs. We included data on mean lifetime clutch sizes for 6256 individuals (*µ* = 3.83; SD = 1.129) and mean initiation dates for 5207 individuals (*µ* = 146.45; SD = 5.6; Julian Day) For individuals lacking data on within‐year measures of reproduction, survival and breeding probability for a given year was simply modeled as the mean plus the annual residual for the purposes of the logistic regression.

### 2.1 Data analysis

#### 2.1.1 Estimating breeding, survival, and encounter probabilities

We estimated apparent annual survival and breeding probability using robust design models with both primary and secondary occasions (Kendall & Nichols, 1995; Riecke et al., 2018). The robust design uses information from encounters during secondary occasions to estimate encounter probability for the primary occasion (breeding season) conditioned on being present (Kendall & Nichols, 1995). This additional information allows the estimation of presence or temporary absence, breeding or nonbreeding in our system (Kendall & Nichols, 1995). While we report apparent annual survival, fidelity of experienced breeders to TRC is nearly 1 (Sedinger et al., 2008) so apparent annual survival approximates true annual survival and we refer to our estimates as annual survival throughout. Primary occasions were the time period in which individuals could be encountered, which we defined as the entire summer breeding season from May to July each year. Secondary occasions were distinct periods within the primary occasion, consisting of the nesting period, a period of three weeks following hatch, and the adult remigial molt, described above. Secondary occasion encounter data were represented by *y_i_*_,_ *_t_*_,_ *_k_*, where *i* = 1,…, *n* indicated the individual, *t* = 1,…, *T* indicated the primary occasion, and *k* = 1, 2, 3 indicated the secondary occasion. The robust design assumes the population is demographically closed between secondary occasions within a primary occasion (Kendall & Nichols, 1995). Previous research has shown that failed breeders depart between nesting and later secondary occasions but simulations of this lack of closure and its effects found only minor (<2%) bias in breeding probability estimates (Sedinger et al., 2001). We assumed the data arose from a Bernoulli distribution with the probability of success equal to the encounter probability of the secondary occasion (*p_t_*_,_ *_k_*) and conditional on the individual's sampling availability (*π_i_*_,_ *_t_*) and latent state (alive or dead, *z_i_*_,_ *_t_*)

$$y_{i,t,k}∼\left\{\begin{array}{cc} \text{Bernoulli}\;(p_{t,k}), & z_{i,t}\pi_{i,t}=1\\ 0, & \text{otherwise} \end{array}\right..$$

We defined an individual's sampling availability as a Bernoulli random variable, conditional on the individual's latent state and breeding probability (*γ_i_*_,_ *_t_*),

$$\pi_{i,t}∼\left\{\begin{array}{cc} \text{Bernoulli}\;(\gamma_{i,t}), & z_{i,t}=1\\ 0, & \text{otherwise} \end{array}\right..$$

We modeled an individual's latent state using a Bernoulli distribution and the individual's annual survival probability (*φ_i_*_,_ *_t_*), conditional on the individual's previous latent state

$$z_{i,t}=\left\{\begin{array}{cc} \text{Bernoulli}\;(\varphi_{i,t}), & z_{i,t‐1}=1\\ 0, & \text{otherwise} \end{array}\right..$$

#### 2.1.2 Relationships between clutch size and initiation date, and annual survival and breeding probability

**FIGURE 1:**
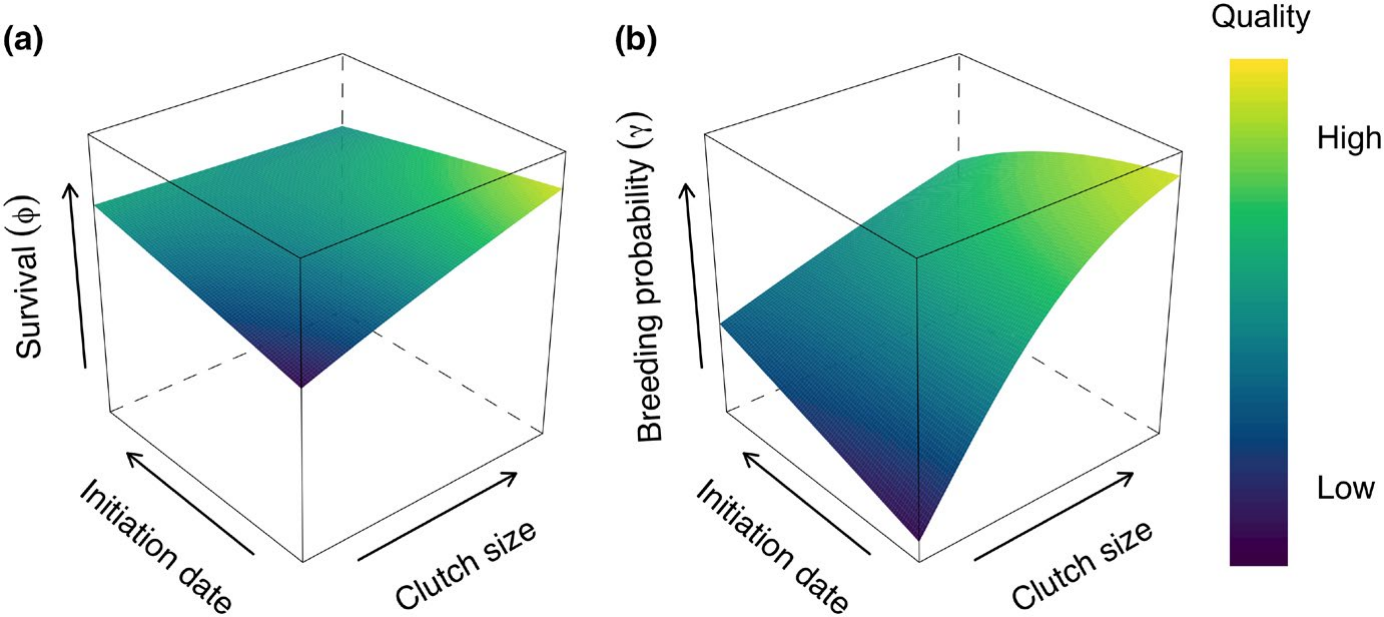
Theoretical gradients of survival (a) and breeding probability (b). Under the demographic buffering hypothesis, long‐lived species should have little within‐population variation in survival across gradients of individual quality. Breeding probability, however, should show substantial variation, with high quality having much higher breeding probabilities than low‐quality individuals

Within the same model, we estimated the effects of clutch size (*κ_i_*) and initiation date (*δ_i_*) on annual survival (*φ_i_*_,_ *_t_*) and breeding probability (*γ_i_*_,_ *_t_*) using the lifetime means of observed individual clutch sizes and initiation dates. We *z*‐standardized clutch size and initiation date within years and then took the mean across years for each individual to produce a value representing average within‐year reproductive energy allocation. These measures essentially placed individuals on a “quality” gradient, where individuals laying larger and earlier clutches were considered higher “quality,” as on average they were better able to acquire nutrients for annual reproductive allocation, and theoretically should have increased survival and breeding probability (Figure 1). Our analyses did not assess the potential for trade‐offs between annual survival or breeding probability and reproduction in a particular year, as this has been considered elsewhere (Leach et al., 2019). We assessed the relationships between among‐year reproduction (mean lifetime clutch size and initiation date) and the demographic rates (annual survival and breeding probability) using a generalized linear model, with a logit link function. Temporal variability was also included in candidate models (*ε*) for each year

$$\text{logit(}\varphi_{i,t})=\beta_{0}+\beta_{1}\kappa_{i}+\beta_{2}\delta_{i}+\beta_{3}\kappa_{i}\delta_{i}+\varepsilon_{\phi ,t}$$

$$\text{logit(}\gamma_{i,t})=\alpha_{0}+\alpha_{1}\kappa_{i}+\alpha_{2}\delta_{i}+\alpha_{3}\kappa_{i}\delta_{i}+\varepsilon_{\gamma ,t}.$$

The interaction between clutch size and laying date assessed the effect on annual survival or breeding probability of deviating from the mean relationship between clutch size and laying date. Such deviations (e.g., clutches that are larger than predicted for a particular laying date) allowed us to assess the potential for trade‐offs between investments in eggs and annual survival or breeding probability, after controlling for laying date. That is, did individuals suffer a reduction in annual survival or breeding probability if they produced larger clutches than predicted by the clutch size‐laying date trend?

#### 2.1.3 Parameter model

Model priors for the above parameters are as follows.

$$\beta_{0}∼\text{logit(Beta(}8.5,\;1.5))$$

$$\alpha_{0}∼\text{logit(Beta(}7.2,\;2.8))$$

β∼Normal(0,100I)

α∼Normal(0,100I)

$$p_{t}∼l\text{ogit(Normal(}\mu_{j},\;\sigma_{p,j}))$$

$$\mu_{j}∼\text{Normal(0,}\;0\text{.01)}$$

$$\sigma_{p,j}∼\text{Uniform(0,}\;5)$$

ε∼Normal(0,σ)

σ∼Uniform(0,5)

We performed analyses in JAGS (Plummer, 2003) using the R (R Core Team, 2018) package *jagsUI* (Kellner, 2018). We sampled two chains for 15,000 iterations, with a burn‐in of 10,000, and a thinning rate of 2. We report the posterior medians, 95% credible intervals, and *ξ* (the proportion of the posterior distribution on the same side of 0 as the mean, Plummer, 2003). The Gelman–Rubin statistic (*R*) was calculated for all parameters to assess model convergence and traceplots for regression coefficients were examined. To assess goodness of fit, we calculated a Bayesian *p*‐value using a targeted discrepancy function (Conn et al., 2018). MCMC chains for all parameters converged ($\hat{R}$ < 1.01). The Bayesian *p*‐value was .516, indicating good model fit.

## 3 RESULTS

Mean annual survival of breeding females was 0.825 (95% CRI: 0.807–0.843, SD = 0.009) across all years and individuals. Annual survival was not correlated with initiation date (*β*_2_ = −0.003; 95% CRI = −0.041 to 0.035; SD = 0.019; *ξ* = 0.558). However, model estimates did provide evidence for a modest positive relationship between annual survival and clutch size (*β*_1_ = 0.03; 95% CRI = −0.013 to 0.073; SD = 0.022; *ξ* = 0.916). Mean breeding probability was 0.818 (95% CRI: 0.754–0.868, SD = 0.03) across all years and individuals. Breeding probability was strongly related to both clutch size and laying date (Table 1). Breeding probability was positively related to clutch size (*α*_1_ = 0.447; 95% CRI = 0.33–0.569; SD = 0.062; *ξ* = 1.000) and negatively related to initiation date (*α*_2_ = −0.115; 95% CRI = −0.196 to −0.036; SD = 0.041; *ξ* = 1.000). Overall, relationships between annual survival and breeding probability, and clutch size were consistent with the hypothesis that fitness traits covary along an axis of individual quality, where individuals that laid larger, earlier clutches were also more likely to breed in future years.

**TABLE 1:** Estimates of *β* and *α* parameters for the relationships between average lifetime clutch size, nest initiation date, and their interaction on apparent survival and breeding probability

Parameter	*μ*	*σ*	2.5% CRI	97.5% CRI	*ξ*
*β*_0_	0.825	0.009	0.807	0.843	1
*β*_1_	0.03	0.022	−0.013	0.073	0.916
*β*_2_	−0.003	0.019	−0.041	0.035	0.558
*β*_3_	0.031	0.025	−0.017	0.08	0.894
*α*_0_	0.818	0.3	0.754	0.869	1
*α*_1_	0.447	0.062	0.33	0.569	1
*α*_2_	−0.115	0.041	−0.196	−0.036	0.998
*α*_3_	−0.111	0.05	−0.212	−0.012	0.986

Note

Estimates come from hierarchical Bayesian robust design models that used reproductive and capture–recapture data collected from female black brant breeding on the Yukon‐Kuskokwim Delta, Alaska (1988–2014). Parameters include the mean (*µ*), standard deviation (*σ*), 95% credible intervals (CRI), and the proportion of the posterior distribution on the same side of 0 as the mean (*ξ*). Note that estimates for *β*_0_ and *α*_0_ are logit‐transformed.

**FIGURE 2:**
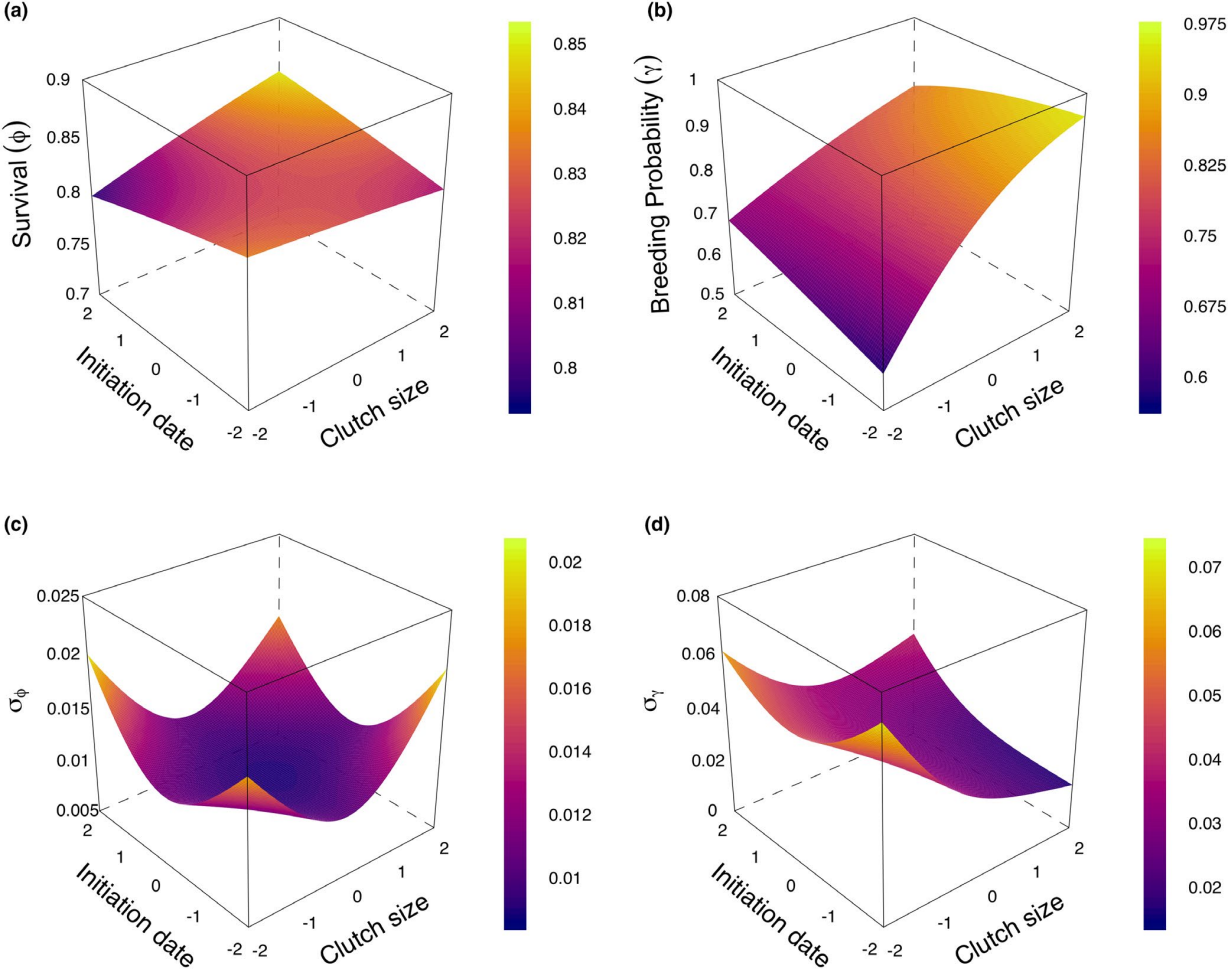
Predicted survival (a) and breeding probability (b), and *σ* values of predicted survival (c) and breeding probability (d), using clutch size, initiation date, and clutch size/initiation date interaction for female black brant breeding on the Yukon–Kuskokwim River Delta, Alaska (1988—2014). Surfaces were predicted from linear models associated with survival and breeding probability and associated parameter estimates described in the Sections 2 and 3

Annual survival had a weak positive relationship with the interaction between clutch size and laying date (*β*_3_ = 0.031; 95% CRI = −0.017 to 0.08; SD = 0.025; *ξ* = 0.894), suggesting that annual survival is largely buffered from variation associated with other fitness traits. Breeding probability was strongly negatively related to the interaction between clutch size and initiation date (*α*_3_ = −0.111; 95% CRI = −0.212 to −0.012; SD = 0.05; *ξ* = 0.986; Figure 2b,d). The predicted response surface was relatively flat for clutch sizes larger than the mean for a particular laying date. In contrast, females producing clutches smaller than the mean tended to have substantially lower breeding probabilities. For example, mothers with clutch sizes two standard deviations below the mean had a breeding probability of 0.637 (~2 eggs; 95% CRI = 0.541–0.733), about 30% less than mothers with clutch sizes two standard deviations above the mean (~5 eggs; *µ* = 0.912; 95% CRI = 0.865–0.944).

**FIGURE 3:**
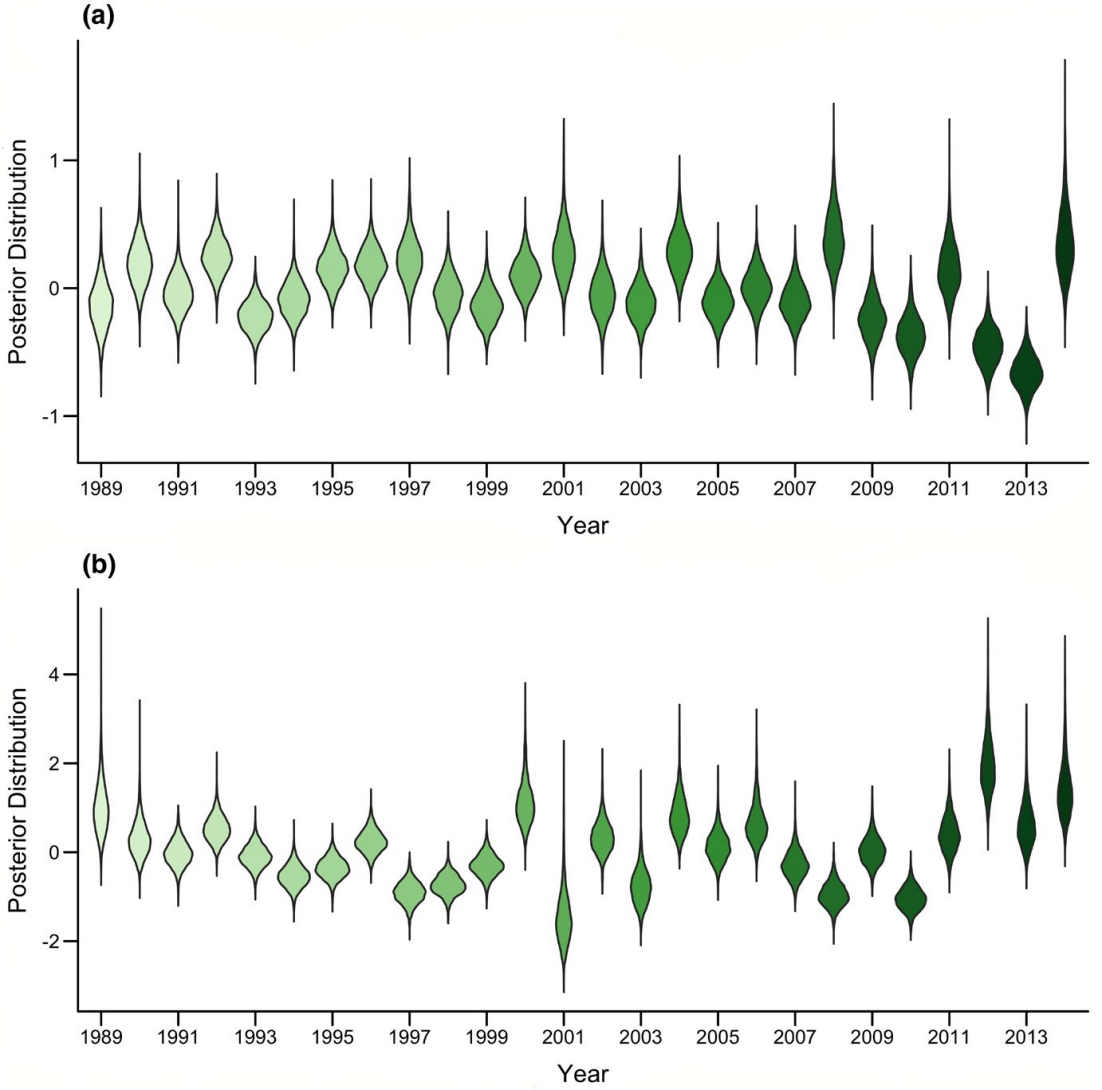
Posterior distributions of temporal variability parameters for survival (*ε_t_*_,_ *_φ_*; a) and breeding probability (*ε_t_*_,_ *_γ_*; b) included in linear regression models

Temporal variability in survival (*ε_t_*_,_ *_φ_*; Figure 3a) was lower than that for temporal variability in breeding probability (*ε_t_*_,_ *_γ_*; Figure 3b). This is consistent with predictions for long‐lived species under the demographic buffering hypothesis (Rotella et al., 2012).

## 4 DISCUSSION

Our results identify an axis of positive relationships among traits positively related to fitness in brant. Individuals producing the earliest (below the 10^th^ percentile), and largest clutches (above the 90^th^ percentile), which maximize current reproductive fitness, had a 19% higher probability of breeding than females producing the latest (above the 90^th^ percentile) and smallest clutches (below the 10^th^ percentile; Figure 2b). Thus, females that experience the greatest reproductive fitness in one year are more likely to do so throughout their lifetimes because they have a greater probability of breeding. The interaction term revealed additional structures in the relationship among clutch size, laying date, and breeding probability. Females producing smaller and earlier clutches than the mean had a reduced probability of breeding (Figure 2b), while there was a substantially weaker relationship for females producing clutches larger and later than the mean and breeding probability (Figure 2b).

The relationship between clutch size and breeding probability is consistent with results from experimental reduction of clutches in brant (Leach et al., 2019) and observations of higher probabilities of breeding in the year after a successful breeding attempt (Sedinger et al., 2011). One likely mechanism for these patterns is the positive association between family size and social status in winter flocks of geese (Black et al., 1992; Raveling, 1970), including brant (Poisbleau et al., 2006). These carry‐over effects of reduced breeding probability following the production of a small clutch have a larger effect on fitness than just reduced breeding probability the next year. Once an individual has skipped breeding, their probability of returning to the breeding population is about half of that for a female that did nest (Sedinger et al., 2008).

The predicted response surface relating annual survival to clutch size and laying date was much flatter than that for breeding probability with annual survival ranging from 0.78 to 0.85 across the range of clutch size‐laying date combinations (Figure 2a). The shape of the surface induced by the interaction between clutch size and laying date suggested weak destabilizing selection away from the mean clutch size‐laying date line; females producing large, late clutches and small, early clutches tended to survive at slightly higher rates than females producing large, early clutches or small, late clutches, respectively. This trend would indicate a slight fitness benefit incurred by deviating from the established clutch size/laying date relationship followed by brant and other long‐lived capital breeders, which is presumably counterbalanced by the optimization of the clutch size‐laying date relationship. However, we urge caution in interpreting this result because evidence for the interaction was modest and the overall surface was relatively flat.

The patterns we report here are consistent with the hypothesis that early environment and maternal effects have an important influence on adult fitness in brant, though they are not the only factors driving individual heterogeneity in fitness. These effects (Cooch et al., 1991; Larsson & Forslund, 1991; Sedinger, Flint, et al., 1995; Sedinger et al., 2004) help drive adult body size and reproductive fitness in several goose species. Hatch date plays a prominent role in such variation because early hatching goslings grow more rapidly (Cooch et al., 1991; Lindholm et al., 1994; Sedinger & Flint, 1991), resulting in larger adult body sizes (Larsson & Forslund, 1991). Further, the size of eggs produced by female geese explains some variation in gosling size at fledging (Acevedo et al., 2020). While much of the relevant variation appears heritable (Larsson & Forslund, 1992), the general lack of response to apparently strong selection for larger body sizes (Riecke, 2020) and associated reproductive variables suggests that either such heritability does not have a substantial additive genetic basis, or there are unknown associated negative genetic covariances (Hoffmann & Merilä, 1999; Larsson, 1993; Price & Liou, 1989). We generally found that fitness‐related life‐history traits were positively correlated, consistent with a primarily non‐additive genetic explanation for this variation, though our analysis was not exhaustive.

**FIGURE 4:**
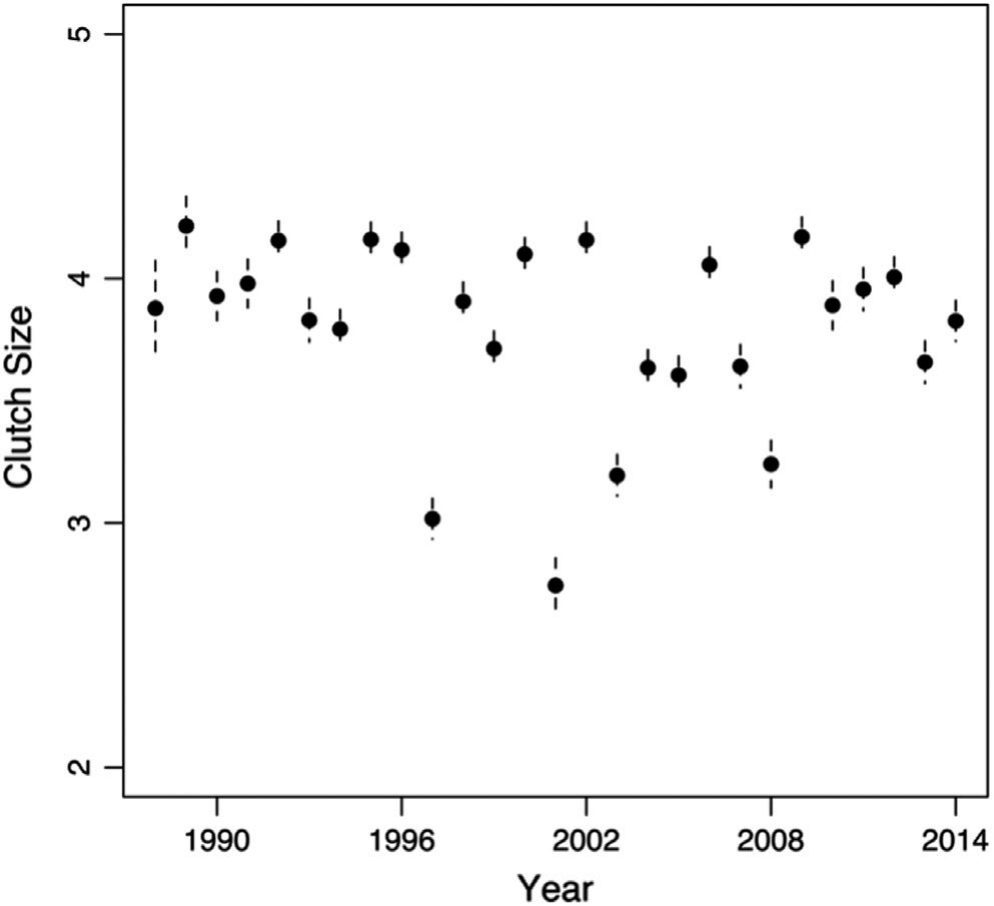
Yearly mean and 95% confidence intervals of predicted clutch sizes of female black brant breeding on the YKD in Alaska from 1988 to 2014. Predicted clutch sizes for each year were calculated using a frequentist linear regression

Over the past three decades, clutch size for brant has remained relatively constant, indicating that clutch size has not responded to selection (Figure 4). We propose that an increase in clutch size would require an increase in adult body size to reduce the effects of a trade‐off between number and quality of offspring that exists currently (Acevedo et al., 2020). Differences in environmental conditions during growth largely determine heterogeneity in body size and individual quality which exists within (Riecke et al., 2018; Sedinger & Chelgren, 2007) and among (Lohman et al., 2019) cohorts. Previous work has shown the importance of environmental conditions during growth; nutrient availability during the breeding season can affect first‐year survival (Sedinger & Chelgren, 2007), recruitment of young into the breeding population (Lindström, 1999; Sedinger et al., 2004), and their fitness as an adult (Sedinger et al., 1995). Thus, variation in growth conditions produces variation in gosling size at fledging (Cooch et al., 1991; Sedinger et al., 2004) and adult size and life‐history traits (Douhard et al., 2014; Sedinger, Flint, et al., 1995). Foraging conditions for brant goslings have declined through time (Lohman et al., 2019; Sedinger et al., 2016) resulting in a general decline in gosling size at fledging (Lohman et al., 2019). A strong positive association between gosling size and first‐year survival (Sedinger & Chelgren, 2007), however, implies that a declining mean in gosling size has resulted in increasingly strong selection acting on brant cohorts through time. In fact, Leach, Ward, et al. (2017) reported that first‐year survival declined from 70% to ≤20% from the early 1990s to the early 2010s. This pattern of selection against small individuals after their first summer has resulted in only relatively small declines in adult body size (Riecke, 2020) and no clear trend in clutch size (Figure 4), consistent with the idea that much of the variation results from environmental or maternal origins. Nevertheless, we cannot rule out negative pleiotropy (Sinervo & Svensson, 1998) to explain the relatively weak response to selection.

Our findings are consistent with the demographic buffering hypothesis for long‐lived, iteroparous organisms. This hypothesis states that because population dynamics and individual fitness are more sensitive to annual survival than reproduction (Gaillard et al., 1998; Rotella et al., 2012; Schmutz et al., 1997), selection has tended to canalize adult annual survival, resulting in reduced variance in survival relative to other demographic traits. For example, the nearly sevenfold greater range of variation in breeding probability compared to annual survival of adult female brant (Table 1, Figure 2a,b) complies with expectations under demographic buffering. Moreover, the relatively flat selection gradient on clutch size, owing to variation in survival (Table 1, Figure 2a), would follow from a considerable lack of variation in innate survival from the brant population (Gaillard et al., 1998; Sæther & Bakke, 2000).

Understanding links between within‐ and among‐year reproductive trade‐offs could help better predict population trends moving forward for brant and other long‐lived organisms. Our results provide further evidence for the demographic buffering hypothesis and demonstrate substantial individual heterogeneity in fitness. This heterogeneity is reflected both in the general positive covariation among several components of fitness and in carry‐over effects of reproductive tactics from one breeding season to the next. This work, in conjunction with earlier research (Douhard et al., 2014; Monaghan, 2007; Sedinger et al., 2004), indirectly provides support for the importance of early growth conditions as regulators of individual fitness and population growth rates.

## CONFLICT OF INTEREST

There are no conflicts of interest to declare.

## AUTHOR CONTRIBUTION

**Madeleine G. Lohman:** Conceptualization (equal); Formal analysis (equal); Visualization (lead); Writing‐original draft (lead); Writing‐review & editing (equal). **Thomas V. Riecke:** Conceptualization (equal); Data curation (equal); Formal analysis (equal); Funding acquisition (equal); Methodology (equal); Resources (supporting); Writing‐review & editing (equal). **Perry J. Williams:** Supervision (supporting); Writing‐review & editing (equal). **James S. Sedinger:** Conceptualization (equal); Data curation (lead); Funding acquisition (lead); Investigation (lead); Project administration (lead); Resources (lead); Supervision (lead); Writing‐review & editing (equal).

### OPEN RESEARCH BADGES

This article has earned an Open Data Badge for making publicly available the digitally‐shareable data necessary to reproduce the reported results. The data is available at [https://doi.org/10.5061/dryad.z08kprrdg].

## Data Availability

Data are available on the Dryad Digital Repository (https://doi.org/10.5061/dryad.z08kprrdg).
